# Double-Crosslinked Polyurethane Acrylate for Highly Conductive and Stable Polymer Electrolyte

**DOI:** 10.3390/polym12112557

**Published:** 2020-10-31

**Authors:** Han-Na Kim, Kyung-Geun Kim, Yeon Uk Jeong, Sung Yeol Kim

**Affiliations:** 1 School of Mechanical Engineering, Kyungpook National University, Daegu 41566, Korea; gks9575@naver.com (H.-N.K.); kimkg.me@gmail.com (K.-G.K.); 2 School of Materials Science and Engineering, Kyungpook National University, Daegu 41566, Korea; jeong@knu.ac.kr

**Keywords:** polyurethane acrylate, polymer electrolyte, ionic conductivity, stability

## Abstract

High ionic conductivity and good stability are major factors that influence the use of polymer electrolytes in electrochemical storage and conversion devices. In this study, we present polyurethane acrylate (PUA) membranes having enhanced ionic conductivity and swelling stability by double crosslinking the polyurethane (PU) and polyacrylate (PA) compartments. The crosslinking agent concentration was varied to control their mechanical properties, swelling stability, and ionic conductivity. Under optimum conditions, the electrolyte uptake of the double-crosslinked PUA membranes without notable defects was 245%. The maximum ionic conductivity of these membranes reached 9.6 mS/cm, which was higher than those with respect to most of the previously reported PUA- or PU-based polymer electrolytes.

## 1. Introduction

Polymer electrolytes based on polyurethane acrylate (PUA) have been investigated for usage in electrochemical energy storage applications because of their high ionic conductivity, excellent mechanical resilience, and easily controllable physical properties [1,2,3,4,5]. PUA is a complex of polyurethane (PU) and polyacrylate (PA). The ionic conduction of PUA can be attributed to the PU compartments; when polyols [e.g., poly(ethylene glycol) (PEG)] were used as the soft segment of the PU compartment, the solid PUA membranes exhibited moderate ionic conductivity in the order of 10^−3^ to 10^−2^ mS/cm at room temperature [3,6,7,8]. In the case of the hard segment of the PU compartment, a chain extender molecule, dimethylolpropionic acid (DMPA) was found to promote single-ion conduction by affecting the interaction between the carboxylic pendant and cation in the electrolyte [3,5]. 

In general, the ionic conductivity of polymers can be significantly enhanced by the gelation of the polymers using liquid plasticizers (e.g., propylene carbonate) [9]. The conductivity of PUA membrane increased 10- to 20-fold compared with that of the solid one before gelation [3,8]. This increase can be attributed to the additional ionic conduction occurring through the liquid phase in the gel, which is independent of the segmental motion of the polymer chain [10]. However, the gelation often causes dimensional instability, such as crack formation and subsequent fragmentation of the gels, if the polymer matrix is not appropriately crosslinked because of the large volume increase.

The PA compartment of PUA plays the main role in preventing such an instability; the crosslinked network of PA, which is typically formed by the polymerization of the acrylate end group of PU, enables the PUA matrix to resist osmotic pressure and other external forces [4]. Recently, we reported a gel polymer electrolyte based on a PUA membrane crosslinked solely with the PA compartment [5]. Its ionic conductivity increased with increasing LiClO_4_ concentration from 20 to 60 wt % of the polymer matrix, becoming a maximum of 3.72 mS/cm. PUA membranes with higher salt concentrations (>60 wt %) were prone to break and their surface became nonconductive with the precipitated salt LiClO_4_. Similar trends were also reported for other gel polymer membranes [11]. Therefore, to advance PUA membranes, electrolyte uptake capacity and dimensional stability should be enhanced simultaneously.

One method to achieve this objective would be the dual crosslinking of PUA: crosslinking of both the PU and PA networks. To the best of our knowledge, all the previously reported PUA electrolytes were based only on the crosslinking of PA [4,5,12]. The additional crosslinked network of PU would not only enhance dimensional stability but also affect ionic conductivities, because polymer crosslinking generally decreases the degree of polymer crystallinity and influences its affinity to the solvent for gelation [13]. Many studies have been reported in that double crosslinking of other polymers (e.g., polyacrylic acid) improved their mechanical properties such as resilience [14], as well as toughness and strength [15]. A recent study has shown that dual crosslinking of poly(propylene oxide) results in an elastic lithium-ion conductor [16]. 

In this study, we report the fabrication of double-crosslinked PUA membranes using PEG, DMPA, and a PU crosslinking agent, i.e., trimethylolpropane (1,1,1-tris(hydroxymethyl) propane; TMP). The TMP/PEG ratio was controlled to study the effects of PU crosslinking on the PUA electrolyte properties. The tensile tests showed that the Young’s modulus of PUA increased with the increasing TMP/PEG ratio, indicating a high degree of PU crosslinking. The swelling tests of LiPF_6_/ethyl carbonate (EC)/dimethyl carbonate (DMC) showed that double-crosslinked PUA membranes (TMP/PEG > 0) remained stable, whereas PUA membranes solely based on PA crosslinking was prone to fragmentation and surface cracking. In addition, the ionic conductivity increased up to 9.6 mS/cm when the TMP/PEG ratio was increased to 0.08 (i.e., TMP/PEG = 0.04/0.5). This room-temperature conductivity value was approximately twice that of the PUA membranes without TMP, confirming that double crosslinking of the PUA enhanced its ionic conductivity as well as dimensional stability. 

## 2. Materials and Methods

### 2.1. Materials

DMPA, TMP, PEG (M_n_ = 3350 g/mol), pentaerythritol triacrylate (PETA), dibutyltindilaurate (DBTDL), 4,4ʹ-methylenebis(cyclohexyl isocyanate), isocyanate (H_12_MDI), and azobisisobutyronitrile (AIBN) were purchased from Sigma-Aldrich (St.Louis, MO, USA). Lithium hexafluorophosphate salt (LiPF_6_), EC, and DMC were obtained from Tokyo Chemical Industry (Kita-ku, Tokyo, Japan) and Daejung Chemical & Metals Co, Ltd (Siheung-si, South Korea). H_12_MDI was dried using 4-Å molecular sieves. Before mixing, PEG was melted at 60 °C and LiPF_6_ and PETA were dissolved in EC/DMC (v:v = 50:50). AIBN (Sigma-Aldrich) was added before casting and curing the PUA membranes.

### 2.2. Fabrication of Gel PUA Membranes 

The fabrication of the gel PUA membranes involved PUA prepolymer synthesis, casting/curing, and swelling. To synthesize a PUA prepolymer, PEG, DMPA, and TMP were added to a four-necked round-bottomed flask equipped with inlets for an external thermometer, a stirrer, nitrogen gas purging, and a heat jacket. The mixture was degassed in vacuum at 85 °C for 1 h and cooled to 55 °C. Subsequently, H_12_MDI/DBTDL was added dropwise to the flask, and the mixture reacted at 85 °C for 3.5 h to form a crosslinked PU prepolymer. The prepolymer was then cooled to 45 °C, and PETA was added dropwise. This reaction mixture was maintained at 45 °C for 15 h to yield the acrylate-terminated prepolymer. After the resulting prepolymer was cooled to 30 °C, 1 M LiPF_6_ in EC/DMC (50:50 vol%) and AIBN were added at 100% and 0.25 wt % of the prepolymer, respectively. The sample was thoroughly mixed for 4 h and then dried at 45 °C for 24 h to remove the bubbles in the membrane. The mixture was then cast and cured at 80 °C for 10 h. The resulting PUA membrane was immersed and swollen in 1 M LiPF_6_ in EC/DMC (50:50 vol%) to obtain PUA membranes in gel form. The optimum concentration was determined to be 1 M to yield PUA polymers with high conductivity (see Supporting Information Figure S1). To investigate the swelling capability of the PUA membranes, the percent electrolyte uptake was measured over time based on the weight gain percentage as follows:

(1) $$solution\;uptake\;\left(\%\right)=\frac{W_{s\;}\;-\;\;W_{d}}{W_{d}}\;\times \;100\;\left(\%\right)$$

where ***W_d_*** is the weight before swelling and ***W_s_*** is the weight after swelling. The membranes were swollen in a closed container to prevent the solvent evaporation, and their weights were measured before and after swelling for 1, 4, 8, and 16 h using a microbalance.

### 2.3. Characterization of PUA Prepolymers and Membranes

The functional groups of the PUA prepolymers were investigated via FTIR spectroscopy (Frontier, PerkinElmer, Waltham, MA, USA). The PUA prepolymers were coated on a zinc selenide crystal to obtain the IR spectra via the attenuated total reflectance technique. The sample spectra were collected in the wavelength range from 500 to 4000 cm^−1^ at a resolution of 4 cm^−1^. The UV–Vis spectra were obtained in the range of 190–1100 nm using a UV–Vis spectrometer (UV-160 Shimadzu, Kyoto, Japan) to determine the transmission characteristics of the PUA membranes. Self-standing PUA membranes before and after swelling in LiPF6 in EC/DMC were used for UV–Vis spectrum measurement. The sample thickness was ~2.0 mm. The tensile properties of the PUA membranes were investigated following a previously reported procedure [17] based on the ASTM D882 standard using a tensile tester (OTT-01S, Oriental TM Corp, Gyeonggi-do, South Korea) equipped with rubber-coated grips and a 3-kgf load cell. The samples were elongated at a rate of 10 mm/min. 

### 2.4. Electrochemical Characterization 

Electrochemical impedance spectroscopy (EIS, VSP, Bio-Logic Science Instruments, Seyssinet-Pariset, France) was used to measure the ionic conductivity of the PUA membranes. The membranes were sandwiched between two metal (e.g., stainless steel) electrodes, and the impedance was measured in the range of 1 MHz to 10 Hz. The ionic conductivity was calculated using the following equation:

(2) $$\sigma {\left(\Omega \cdot cm\right)}^{-1}=\frac{1}{R_{b}}\cdot \frac{t}{A}$$

where ***R_b_*** is the resistance obtained from EIS, t represents the thickness of the polymer membrane (between 0.1 and 2 mm), and ***A*** represents the cross-sectional area of the membrane in contact with the metal.

## 3. Results and Discussion

### 3.1. Preparation of Polymer Membranes 

**Figure 1:**
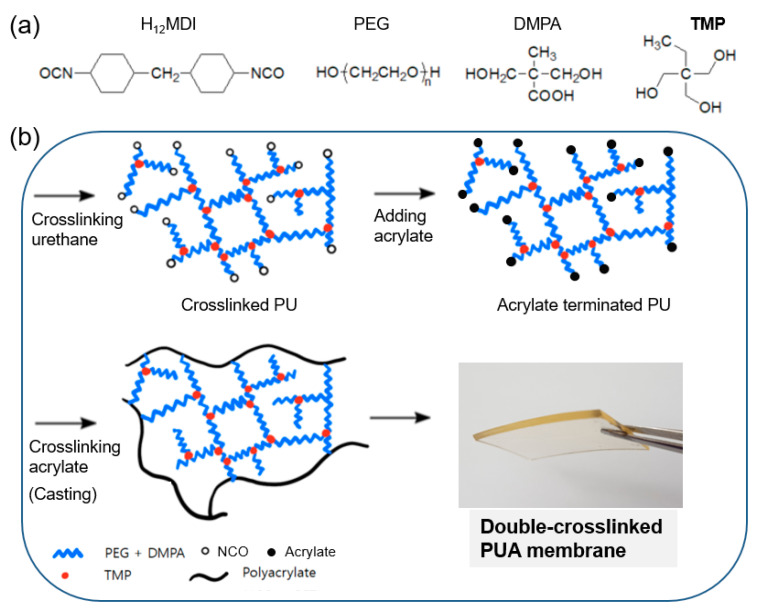
Schematic of the (**a**) chemical components and (**b**) preparation of a double-crosslinked polyurethane acrylate (PUA) membrane.

Figure 1 presents the double-crosslinked PUA membrane components and its preparation processes. First, a crosslinked PU prepolymer was obtained by the reaction of PEG, DMPA, and H_12_MDI with the crosslinking agent TMP. Subsequently, the isocyanate (i.e., NCO) end group was replaced by acrylate to obtain an acrylate-terminated PU prepolymer. The prepolymer was then casted on a flat mold, and acrylate was polymerized and crosslinked to achieve PA crosslinking. The resulting double-crosslinked PUA membrane is shown in Figure 1b. The sample designation and membrane compositions are listed in Table 1. The PUA number represents the molar ratio of TMP to PEG (i.e., the TMP/PEG ratio), which varied from 0.00 to 0.12. The crosslinking agent of PA (i.e., PETA) was fixed, unless stated otherwise. 

### 3.2. Functional Groups of Prepolymers and Their Interactions with Lithium Salt

**Figure 2:**
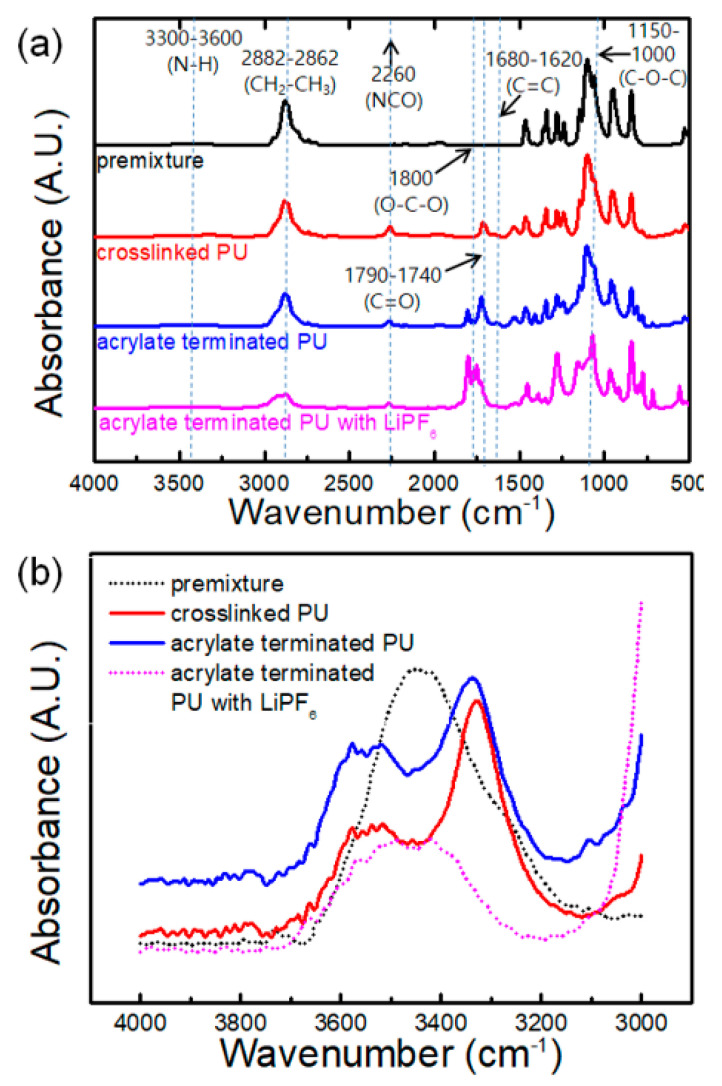
FTIR spectra of the polyurethane (PU) prepolymers at (**a**) 4000–500 and (**b**) 4000–3000 cm^−1^.

Figure 2a shows the overall spectra corresponding to the premixture of PEG, DMPA, and TMP (black line); crosslinked PU prepolymer (red); acrylate-terminated prepolymer with EC/DMC (blue); and acrylate-terminated prepolymer with LiPF_6_ and EC/DMC (magenta). All the samples show peaks at 2882–2886 and 1150 cm^−1^, corresponding to the CH_2_–CH_2_ and C–O–C bonds of PEG, respectively. The peak at 1805 cm^−1^ corresponded to the O–C–O bonds of EC and DMC.

The characteristic bands of NCO, CO, and NH were observed at 2260, 1740–1790, and 3300–3600 cm^−1^, respectively in the spectra of PU prepolymers in Figure 2. As expected, these peaks were not observed in the spectrum of the premixture. The presence of the CO and NH bands in the spectra of the prepolymers confirmed the formation of urethane bonds (CONH). The decreased intensity of NCO peak and the appearance of a C=C peak at 1650 cm^−1^ shown in the spectrum of acrylate-terminated prepolymers without LiPF_6_ and EC/DMC indicated that the NCO group was replaced with acrylate containing a divinyl group.

The –NH stretching region (3100–3800 cm^−1^) was deconvoluted to investigate the molecular interactions between the functional groups and lithium salt (see Supporting Information Figure S2). Three –NH peaks can be observed at 3266–3395 cm^−1^ (peak a), 3436–3516 cm^−1^ (peak b), and 3558–3598 cm^−1^ (peak c), corresponding to the free –NH stretching vibrations, hard–hard segment hydrogen bonding with carbonyl oxygen, and hard–soft segment hydrogen bonding with ether oxygen, respectively [5,18,19]. The incorporation of the Li salt solution resulted in an overall decrease in the –NH peak and a peak shift. The free –NH peak shifts toward a higher wavelength can be attributed to the decrease in N–H bond length because of the interactions of Li^+^ with the lone pair electrons of the N atoms [19]. The peak areas of the free NH and H bonds with carbonyl oxygen increased with increasing Li salt concentration, whereas those of the H bonds with ether oxygen decreased. These results indicate that lithium salt preferably coordinated with the ether oxygen of PEG, resulting in free –NH peaks.

### 3.3. Optical and Mechanical Properties

**Figure 3:**
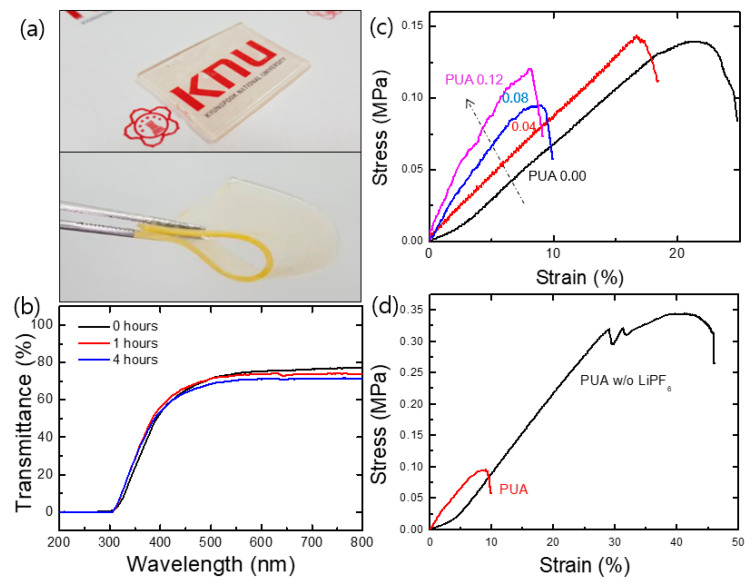
Digital images of the self-standing PUA membranes (thickness: 2.0 mm) showing their (**a**) optical transparency and flexibility and their (**b**) UV–Vis transmittance depending on swelling time. Stress–strain curves of (**c**) PUA membranes and (**d**) PUA 0.08 with and without lithium salt.

Generally, all the double-crosslinked PUA membranes were optically transparent and mechanically flexible (Figure 3a,b). The liquid plasticizer (i.e., EC/DMC) incorporated during the synthesis did not leak after the completion of double crosslinking. The polymer membranes could be easily cut into the desired shape with excellent dimensional stability, exhibiting mechanical flexibility and resilience. The transmittance of the 2-mm-thick PUA membranes was 75% at wavelengths greater than 500 nm and slightly decreased with the swelling time, which can be attributed to the increase in thickness (Figure 3b).

The mechanical properties of the PUA membranes were further investigated using a tensile tester. The stress–strain curves are presented in Figure 3c. The membrane with a high TMP/PEG ratio showed a brittle behavior. Its Young’s modulus was high, as indicated by the steep initial slope, and the fracture strain was low. These results confirm that additional PU crosslinking enhanced the stiffness of the PUA membrane. Because the Young’s modulus is a measure of the degree of crosslinking [20,21], our results are evidence that PUA samples with higher value (i.e., TMP/PEG ratio) have a higher degree of crosslinking. The tensile strength, Young’s modulus, and fracture strain of the PUA membranes range from 90 to 120 kPa, 0.7 to 1.4 MPa, and 10% to 20%, respectively.

Figure 3d shows the tensile curves of a PUA membrane with and without lithium salt. The exclusion of Li salt from the PUA polymers significantly improved the tensile strength (~350%) and the elongation at break (~500%) mainly because the molecular bonding (e.g., hydrogen bonding) among the adjacent PU chains recovered owing to the disappearance of the salt (as mentioned in Section 3.2). In addition, the PA crosslinking agent, PETA, affected the mechanical properties of the PUA membranes. The samples with low PETA concentrations behaved like ductile polymers with a high elongation at break of up to 200%, whereas those with high PETA contents showed brittle behavior (data not shown). These results demonstrate that double-crosslinked PUA membranes with different degrees of crosslinking can be successfully fabricated by controlling the concentration of the crosslinking agents.

### 3.4. Swelling Characteristics of the PUA Membranes: Stability of the PUA Gel Membranes

**Figure 4:**
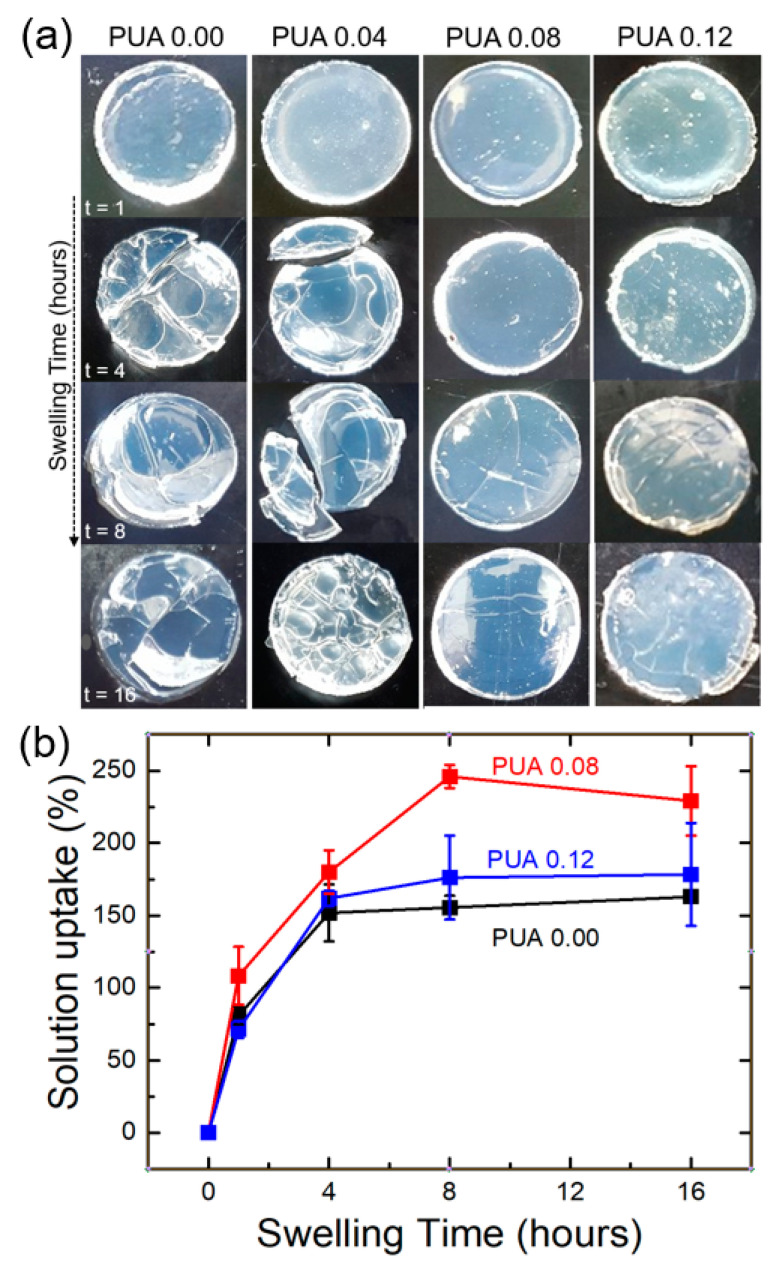
(**a**) Digital images of PUA membranes after swelling for 1 to 16 h (t = 1, 4, 8, 16) in 1 M LiPF_6_ ethyl carbonate (EC)/dimethyl carbonate (DMC) and (**b**) solution uptake of the PUA samples as a function of the swelling time.

All the PUA membranes gradually swelled over time after being immersed in a solution of 1 M LiPF_6_ in EC/DMC. Figure 4a shows the digital images of the PUA membrane during swelling for 16 h. Surface pits and cracks were found on the surfaces of PUA 0.00 and PUA 0.04 after 4 h of swelling. Their sizes and numbers generally increased with time, leading to the fragmentation of the membranes. These pits and cracks confirm that PUA membranes without PU crosslinking are less stable and cannot maintain a high solution uptake with the progress of membrane swelling. However, PUA 0.08 and PUA 0.12 remained relatively intact; their shapes were retained throughout swelling without notable pits and fragmentation, demonstrating that double crosslinking enhanced the stability of the PUA membranes.

The solution uptake of the PUA membranes was quantified as a function of time by measuring the weight percentage gain (Figure 4b). The uptake increased with time (up to 4 or 8 h) and plateaued, indicating it reached equilibrium. PUA 0.08 exhibited the highest uptake over time, with a maximum of 250%, whereas PUA 0.00 showed the smallest uptake, with a maximum of 160%. The limited uptake of PUA 0.00 (sample without PU crosslinking) can be attributed to the loss of the polymer matrix and the subsequent uptake loss, which is supported by the surface pits and cracks observed in these samples. The uptake of PUA 0.12 was smaller than that of PUA 0.08, which can be probably attributed to the small free volumes resulting from the high degree of crosslinking [22]. These swelling test results show that PUA 0.08 has the highest electrolyte uptake capability without showing notable deterioration.

### 3.5. Ionic Conductivity of the PUA Electrolytes

**Figure 5:**
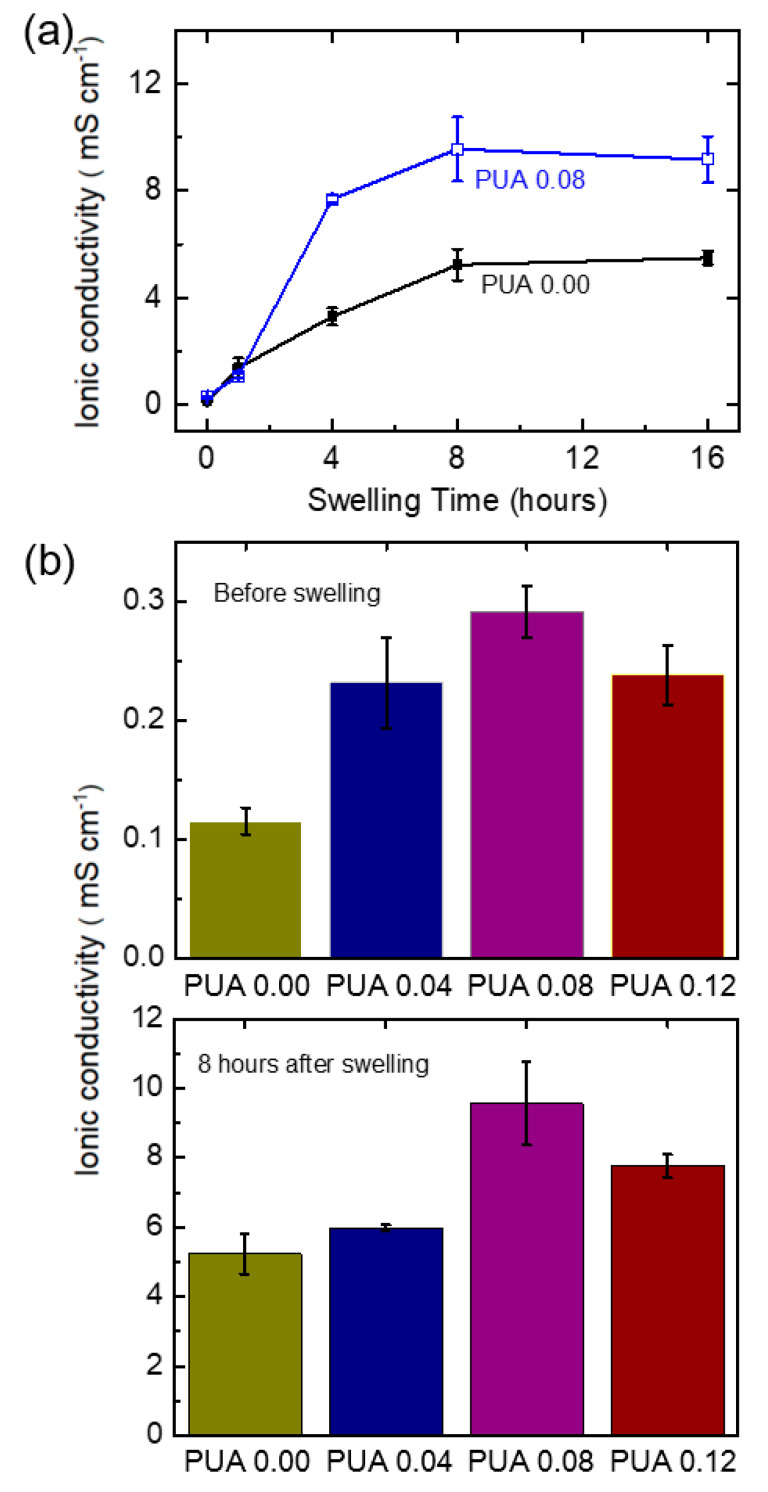
Ionic conductivities of (**a**) PUA 0.08 and PUA 0.00 in the case of different swelling times. PUA membranes (**b**) before swelling and (**c**) after 8 h of swelling.

We investigated the effect of swelling time on the ionic conductivities of the PUA membranes. Figure 5a compares the ionic conductivities of PUA 0.00 and PUA 0.08 as a function of swelling time. In general, the ionic conductivities increased with increasing swelling time up to approximately 8 h and reached an equilibrium value, regardless of the sample compositions. However, the maximum conductivity at equilibrium was dependent on the compositions; PUA 0.08 showed a higher conductivity than PUA 0.00.

Figure 5b compares the ionic conductivities of PUA membranes before swelling (top figure). The ionic conductivity of PUA 0.00 was the lowest (~0.1 mS/cm), and the ionic conductivities of other samples were more than twice higher than that of PUA 0.00, demonstrating the enhancement of ionic conductivity via PUA double crosslinking. This result can be attributed to the structural difference between the membranes—the amorphous structures of the double-crosslinked PUA vs. the ordered structure of the PUA membrane without TMP (see Supporting Information Figure S3). The disordered and less densely packed PUA membranes may facilitate ion movement and improve conductivity.

Figure 5b alsosummarizes the ionic conductivities of the PUA membranes which were swollen for 8 h (bottom figure). It is clear that all the membranes showed more than 20 fold increase in their ionic conductivities after being swollen. This increase is due to the electrolyte uptake observed during the swelling process because high concentrations of charge carriers and solvent contribute to the enhancement of the conductivity. The PUA membranes with high TMP ratios (PUA 0.08 and PUA 0.12) had higher conductivity than those with low TMP ratios (PUA 0.00 and PUA 0.04) possibly due to the high electrolyte uptake. It was difficult to obtain stable PUA 0.00 and PUA 0.04 for performing the conductivity measurements because of the structural instability, as mentioned previously. The highest conductivity of the double-crosslinked PUA electrolytes was 9.6 mS/cm. This conductivity value exceeds those of the highly conductive gel polymer electrolytes reported in the literature, i.e., 8.2 mS/cm for the PU-based gels with clays [2], 4.8 mS/cm for PEO with LiTFSI in succinonitrile [11], and 7.5 mS/cm for the PVDF-based polymer gels [23].

## 4. Conclusions

In this study, we successfully synthesized double-crosslinked PUA membranes with high ionic conductivity and good stability. The ionic conductivity, swelling behavior, and mechanical properties were tuned by controlling the concentration of the crosslinking agent TMP. The Young’s modulus was proportional to the TMP concentration, whereas the ductility was inversely proportional. The electrolyte uptake during the swelling of the PUA membrane was also a function of the TMP concentration and swelling time. The PUA 0.08 membrane had the highest uptake of ~245% at 8 h after swelling, and PUA membranes with low TMPs were mechanically unstable and had a smaller uptake. The highest ionic conductivity in the case of double-crosslinked PUA membranes was 9.6 mS/cm, which exceeds those of most PEO, PUA, and PU electrolytes reported in the literature. Future work will focus on the applicability of these membranes in various electrochemical systems, which require highly conductive, stable, and low-leakage electrolytes.

## Figures and Tables

**Table 1:** Sample designation and polyurethane acrylate (PUA) membrane composition.

Sample Designation	PUA Composition (Molar Ratio)					
PUA TMP/PEG	H_12_MDI	TMP	PEG	TMP/PEG	DMPA	PETA
PUA 0.00	1.1	0	0.5	0.00	0.1	1
PUA 0.04	1.1	0.02	0.5	0.04	0.07	1
PUA 0.08	1.1	0.04	0.5	0.08	0.04	1
PUA 0.12	1.1	0.06	0.5	0.12	0.01	1

## Supplementary Materials

The supplementary materials are available online at https://www.mdpi.com/2073-4360/12/11/2557/s1, Figure S1. Concentration-dependent ionic conductivity of the LiPF_6_ solutions and the prepolymer mixture, Figure S2. Deconvolution of the NH region of (**a**) crosslinked PU, (**b**) acrylate-terminated PU, and (**c**) acrylate- terminated PU with LiPF_6_ in EC/DMC. Peak a: free NH stretching vibration, peak b: hard–hard segment H bonds with the carbonyl oxygen, and peak c: hard–soft segment H bonds with the ether oxygen, Figure S3. Representative DSC curves of PUA membranes.
