# Supplementary material for: Double-Crosslinked Polyurethane Acrylate for Highly Conductive and Stable Polymer Electrolyte
Source: Polymers (Basel). 2020 Oct 31;12(11):2557. doi: 10.3390/polym12112557 (PMC7693480; doi:10.3390/polym12112557)
Supplement: Supplementary file 1 [file polymers-12-02557-s001.pdf]

Supporting Information

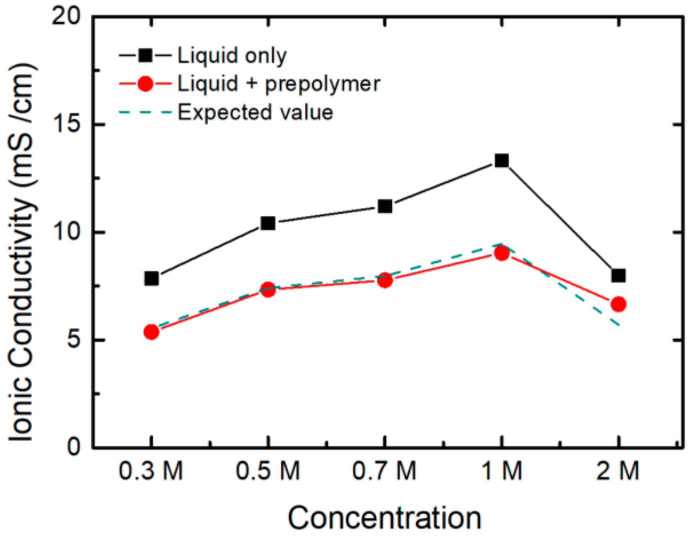

Figure S1. Concentration-dependent ionic conductivity of the $LiPF_6$ solutions and the prepolymer mixture.

Figure S1 shows that the ionic conductivity of the mixture of prepolymers and $LiPF_6$ solution increased when the concentration of $LiPF_6$ was increased from 0.3 to 1 M. However, it decreased at a higher concentration of 2 M mainly due to the strong interactions among the ions.

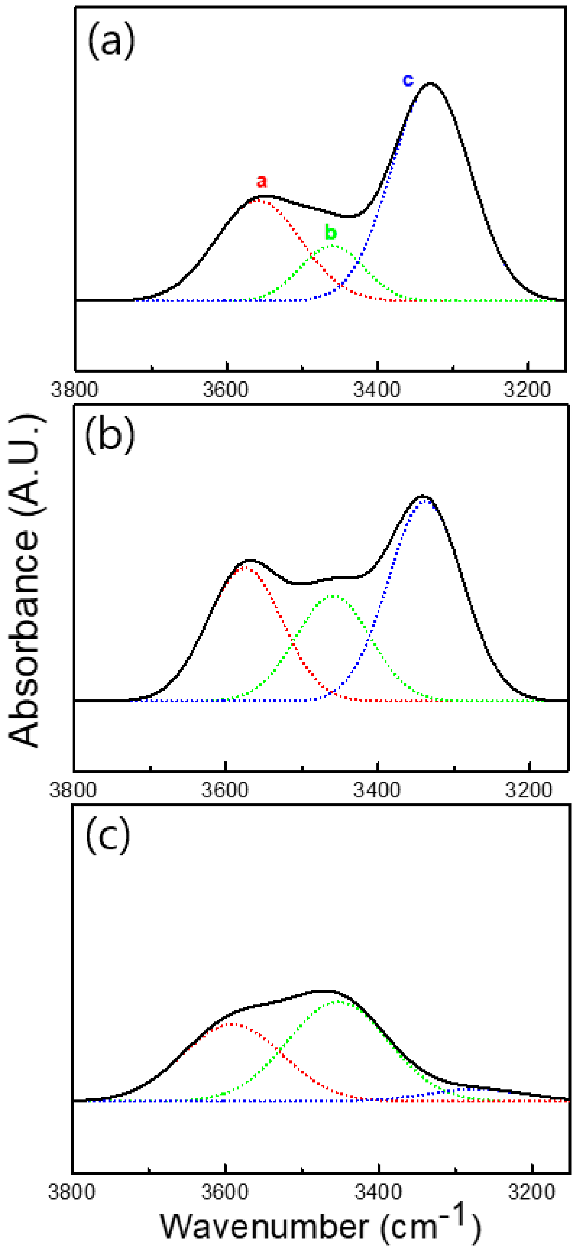

Figure S2. Deconvolution of the NH region of (a) crosslinked PU, (b) acrylate-terminated PU, and (c) acrylate-terminated PU with $LiPF_6$ in EC/DMC. Peak a (red): free NH stretching vibration, peak b (green): hard–hard segment H bonds with the carbonyl oxygen, and peak c (blue): hard–soft segment H bonds with the ether oxygen.

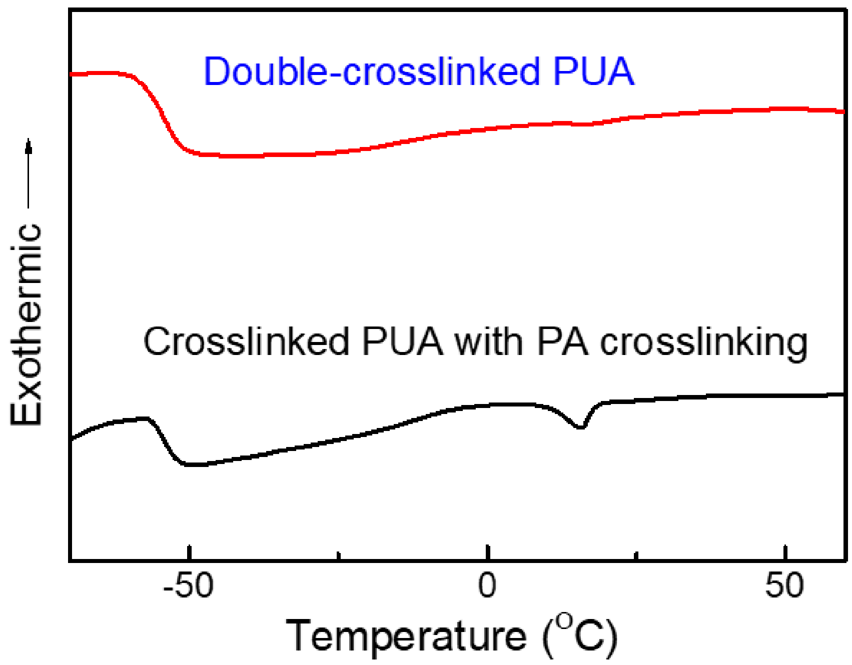

Figure S3. Representative DSC curves of PUA membranes

Double-crosslinked PUA samples showed no endothermic peak corresponding to a melting temperature, indicating that the polymer is amorphous.
